# Predictive factors for complete recovery of post-thrombotic syndrome 6 months after venous recanalization

**DOI:** 10.1016/j.jvsv.2025.102287

**Published:** 2025-07-11

**Authors:** Lina Khider, Costantino Del Giudice, Nicolas Gendron, Chloé Gobert, Benjamin Planquette, Marc Al Ahmar, Guillaume Goudot, Emmanuel Messas, Marc Sapoval, Tristan Mirault

**Affiliations:** a Paris Cité University, INSERM, Paris Cardiovascular Research Centre, Team Endotheliopathy and Hemostasis Disorders, Paris, France; b Vascular Medicine Department, Assistance Publique Hôpitaux de Paris Centre-Université de Paris (APHP-CUP), Paris, France; c Interventional Radiology, Assistance Publique Hôpitaux de Paris Centre-Université de Paris (APHP-CUP), Paris, France; d Hematology Department, Assistance Publique Hôpitaux de Paris Centre-Université de Paris (APHP-CUP), Paris, France; e Respiratory Medicine Department, Assistance Publique Hôpitaux de Paris Centre-Université de Paris (APHP-CUP), Paris, France; f Paris Cité University, PARCC, INSERM U970, Paris, France

**Keywords:** Post-thrombotic syndrome, Chronic venous thrombotic obstruction, Deep venous thrombosis, Stenting, Prediction

## Abstract

**Objective:**

Endovascular recanalization is considered for severely symptomatic patients with post-thrombotic syndrome (PTS) to alleviate symptoms. However, data on complete recovery and its predictors remain limited. This study aimed to assess persistent PTS 6 months after venous recanalization and identify predictive factors.

**Design:**

Single-center retrospective outcome-oriented cohort study.

**Methods:**

We reviewed electronic medical records of patients referred for endovascular venous recanalization between January 1, 2015, and September 30, 2019. Inclusion criteria were PTS defined by a Villalta score of ≥5 or a leg ulcer ≥6 months after a deep vein thrombosis episode. Complete PTS recovery was defined as a Villalta score of <5.

**Results:**

Sixty-seven patients (median age, 40 years; interquartile range, 32-51 years; 78% women; 18% obese) were included. The initial Villalta score was 10 (interquartile range, 7-14). At 6 months, primary and secondary patency rates were 75% and 81%, respectively. Complete recovery was observed in 67% of patients. Multivariate analysis identified the initial Villalta score (odds ratio, 1.36; 95% confidence interval, 1.12-1.65; *P* = .002) and femoral vein obstruction (odds ratio, 3.79; 95% confidence interval, 1.06-13.61; *P* = .041) as predictors of persistent PTS, whereas long-term anticoagulation was not significant.

**Conclusions:**

Endovascular recanalization achieved PTS resolution in two-thirds of patients at 6 months, particularly in those with a low initial Villalta score and no femoral vein obstruction. A risk score incorporating these factors may aid clinicians in patient counseling regarding the expected benefits of the procedure.

**Article Highlights**

• **Type of Research**: Single-center retrospective cohort study
• **Key Findings**: In 67 patients with post-thrombotic syndrome (PTS) undergoing endovascular venous recanalization, primary and secondary patency rates at 6 months were 75% and 81%, respectively. Complete recovery from PTS was achieved in 67% of patients. Predictors of persistent PTS at six months included a high initial Villalta score (odds ratio, 1.36; *P* = .002) and femoral vein obstruction (odds ratio, 3.79; *P* = .041).
• **Take Home Message**: Endovascular venous recanalization can free two-thirds of patients from PTS 6 months after the procedure, particularly those with a low initial Villalta score and no femoral vein obstruction. A predictive risk score could help clinicians in patient counseling regarding the expected benefits.

Post-thrombotic syndrome (PTS) is an important and frequent long-term adverse event affecting 20% to 50% of patients after a deep vein thrombosis (DVT) episode.^1^, ^2^, ^3^, ^4^, ^5^ Characterized by a persistent venous obstruction or a venous reflux, PTS is responsible for chronic limb pain, venous claudication, and swelling and can progress to a worse disability with leg ulcers. PTS significantly impairs patient quality of life and represents a major public health issue.^6^, ^7^, ^8^, ^9^, ^10^ The main risk factors for developing PTS are proximal DVT, especially with iliac involvement,^11^^,^^12^ obesity,^11^^,^^13^ and pre-existing chronic venous disease.^12^^,^^14^^,^^15^ Although therapeutic anticoagulation is essential for preventing recurrent venous thromboembolism, its effect on reducing or preventing PTS remains debated. No drug to date has shown proven efficacy in curing or consistently preventing PTS.^15^ Despite appropriate anticoagulation, ≤50% of patients develop PTS. The effectiveness of conservative treatment with graduated compression stockings remains debated^1^; although venous recanalization has shown promise in symptom relief,^16^ data on complete recovery remain limited.^17^ Currently, only venous compression can slightly reduce the symptoms of PTS. But even with a well-conducted anticoagulant therapy, the persistence of vein obstruction after DVT favors the development of PTS.^18^, ^19^, ^20^ Recanalization of chronic venous thrombotic obstruction by endovascular techniques has shown encouraging results in reducing symptoms of PTS and may be considered for the severely symptomatic patient with iliac vein or vena cava occlusion, according to the American Heart Association and the European Society for Vascular Surgery.^1^^,^^21^^,^^22^ PTS and outflow improvement with a reduction of the Villalta score has been reported after venous recanalization with balloon angioplasty and stenting,^16^^,^^17^^,^^23^, ^24^, ^25^, ^26^, ^27^ but there is much less evidence on complete recovery of PTS. The aim of this study was to evaluate the complete clinical recovery from PTS, defined as a Villalta score of <5 at 6 months after the procedure and to identify its predictive factors. This work could help to identify patients in whom this type of procedure could cure or reduce PTS and thus better inform those who might retain symptoms after venous recanalization.

## Methods

### Patients

This single-center, retrospective study included consecutive patients aged 18 to 75 years who had experienced an acute DVT episode in the femoral or a more proximal vein ≥6 months before the study procedures. According to current anatomical terminology, the term femoral vein refers to the superficial femoral vein, distinct from the common femoral vein, which is reported separately in this study. These patients were eligible for inclusion and were recruited from January 1, 2015, to September 30, 2019, at the Vascular Medicine Department of Hôpital Européen Georges Pompidou (Assistance Publique Hôpitaux de Paris, Paris, France). According to the International Society on Thrombosis and Haemostasis consensus method,^28^ only patients with a Villalta score of ≥5 in the affected limb despite ≥3 months of conservative treatment, consisting of therapeutic anticoagulation and graduated compression stockings (GCS), and with confirmed chronic venous obstruction of the iliac or femoral segment within 60 days before the procedure were included.^28^^,^^29^ Chronic venous obstruction was confirmed in all patients using duplex ultrasound examinations performed by trained vascular specialists. In cases where visualization of iliac or inferior vena cava segments was suboptimal, complementary computed tomography (CT) venography was performed. Magnetic resonance venography was not used in this cohort. This diagnostic strategy aligns with recent expert consensus recommending duplex ultrasound examination as the first-line modality in the evaluation of chronic post-thrombotic lesions.^30^ Although a Villalta score of 5 to 9 is typically classified as mild PTS, we included these patients because many of them reported significant symptoms, especially venous claudication, not captured by the Villalta score. This score does not account for venous claudication, which can be highly disabling and is frequently associated with iliofemoral obstruction. Therefore, patients were considered eligible after multidisciplinary evaluation if symptoms had a substantial impact on daily function.^30^ Patients with extrinsic compression of the iliac vein by the overlying iliac artery (May-Thurner syndrome) were included if associated with chronic thrombotic obstruction. In contrast, patients with extrinsic vein compression due to tumors or arterial aneurysms, as well as those without a 6-month follow-up visit, were excluded. Patients signed informed consent for the procedure and the collection of their data. The study was performed in accordance with the declaration of Helsinki and was approved and referenced 2022-02-06 by the local institutional ethics committee CERAPHP Centre (Paris, France).

### Endovascular procedure

The recanalization procedures were all performed by a senior interventional radiologist (M.S. or C.D.G.). The procedure took place under general anesthesia in an interventional radiology operating room. Complete ipsilateral limb venography was first performed to analyze the venous obstruction, the healthy zone and select the optimal puncture site. Recanalization was primarily performed via bilateral femoral venous approaches, which were used for both iliac and superficial femoral vein occlusions. Venous occlusion was crossed with a stiff hydrophilic guidewire and a support catheter, with an intravenous bolus perfusion of unfractionated heparin (UFH) of 50 UI/kg. After progressive predilation using balloon catheters, extensive stenting was performed between two healthy landing zones. Open-cell nitinol stents —WALLSTENT Endoprosthesis from Boston Scientific (Marlborough, MA), SINUS-XL FLEX from Optimed (Kalamazoo, MI), and Protégé GPS from Medtronic (Minneapolis, MN)—were used, with diameters depending on the anatomic location: 18 to 28 mm for the IVC, 14 to 16 mm for the common iliac, 12 to 14 mm for the external iliac vein, and 10 to 12 mm for the femoral veins. Intravascular ultrasound examination was not available for all procedures during the study period and was used selectively, especially when venography was inconclusive. If a second procedure was required, recanalization also used bilateral femoral venous approaches. Venous obstruction was crossed with a stiff hydrophilic guidewire and a support catheter, with an intravenous bolus of 50 UI/kg UFH. In case of restenosis (refers to a recurrent stenotic lesion identified during a second intervention), after progressive predilation using balloon catheters, intrastent angioplasty was performed with complimentary stenting in case of residual obstruction. Second procedures were considered on a case-by-case basis, in patients with clinical recurrence or worsening of symptoms. Imaging confirmation of in-stent restenosis or reocclusion (by duplex ultrasound examination) was required, and the technical feasibility of reintervention was evaluated before proceeding. Thrombectomy was performed in cases of acute reocclusion diagnosed within 14 days, followed by stenting of the underlying stenosis or any remaining thrombus. Beyond this period, no thrombectomy was attempted. In cases of chronic reocclusion, a standard recanalization procedure—similar to the initial intervention and involving an 0.035” guidewire dilation and intrastent stenting—was scheduled after a minimum delay of 2 months. After the intervention, patients were instructed to wear class II (30-40 mm Hg) knee-high compression stockings for ≥6 months, ideally throughout the day. This approach follows recent expert recommendations and could be extended in cases of persistent symptoms.

### Antithrombotic therapy

Patients were prescribed therapeutic anticoagulation after the procedure with a direct oral anticoagulant (DOAC; rivaroxaban or apixaban), low-molecular-weight heparin, or UFH in case of contraindication to DOAC for ≥6 months. An antiplatelet agent (aspirin or clopidogrel 75 mg once a day) for ≥3 months was added to anticoagulant therapy. Immediately after the procedure and during the first postoperative night, patients had external pneumatic compression. Patients were advised to wear knee-high GCS (30-40 mm Hg) after discharge.^30^

### Follow-up

Patients underwent clinical and duplex ultrasound evaluations at 1 month, 6 months, and annually after the procedure. Duplex ultrasound examinations included assessment of flow direction, respiratory variation, and intrastent velocity. CT venography was used in selected cases when duplex ultrasound findings were inconclusive or inconsistent with clinical presentation.

### Data collection and outcome measures

Data were abstracted from the patient's electronic medical records (DxCare, Dedalus France, Le Plessis Robinson, France) by two independent abstractors (L.K. and C.G.) who reviewed procedural, imaging, and laboratory reports, as well as clinical notes. Regarding the definition of sequelae, we grouped under the term “obstruction” stenoses defined by a reduction in caliber of >50% and venous occlusions.^30^ Follow-up visits collected patency assessment of the treated veins by ultrasound examination and clinical evaluation of the severity of PTS according to the Villalta score: scores ranging from 5 to 9 indicated mild PTS, scores of 10 to 14 indicated moderate PTS, and scores of ≥15 indicated severe PTS.^29^

The primary outcome was the absence of PTS defined by a Villalta score of <5 at 6 months after the procedure. Secondary outcomes were absence of PTS, primary and secondary patency at 1 and 6 months after the procedure, and at the end of follow-up.

Patency was evaluated on color duplex ultrasound examination of the lower limb. Immediate procedural success was defined as successful recanalization on the venography at the end of the procedure defined by the reperfusion of the occluded segment with rapid flow of contrast agent and disappearance of venous collaterals. Primary patency was defined as the percentage of patients with freedom from target lesion recanalization and thrombotic occlusion. Secondary patency was defined as patency after the intervention for complete stent occlusion, that is, when the stent is reopened after a total thrombosis or occlusion, restoring flow. Stent thrombosis was defined as complete stent occlusion or a flow-limiting thrombus within the stent, diagnosed by duplex ultrasound examination or CT venography.^31^

Hemorrhagic complications were also recorded and classified as minor, clinically relevant nonmajor, and major according to the International Society on Thrombosis and Haemostasis classification.^32^^,^^33^

### Statistical analysis

Continuous variables are presented as median (interquartile range [IQR], 25th to 75th percentiles) and compared using Mann-Whitney rank test. Discrete variables are presented as number and percentage and compared using the χ^2^ test or Fisher's exact test, when appropriate. Multivariate analysis was performed using linear logistic regression to calculate odds ratio (OR) and 95% confidence interval (95% CI) with PTS at 6 months as the dependent variable and the significant independent variables from the univariate analysis ie initial Villalta score, long-term therapeutic anticoagulation indication, and femoral vein obstruction within the equation. A risk score was computed based on the OR of significant parameters associated with the persistence of PTS at 6 months. Sensitivity and specificity were computed using receiving operative curve. A *P* value of <.05 was considered significant. All analyses were performed with SPSS version 13.0 (IBM Armonk, NY).

## Results

### Patient characteristics

**Fig 1:**
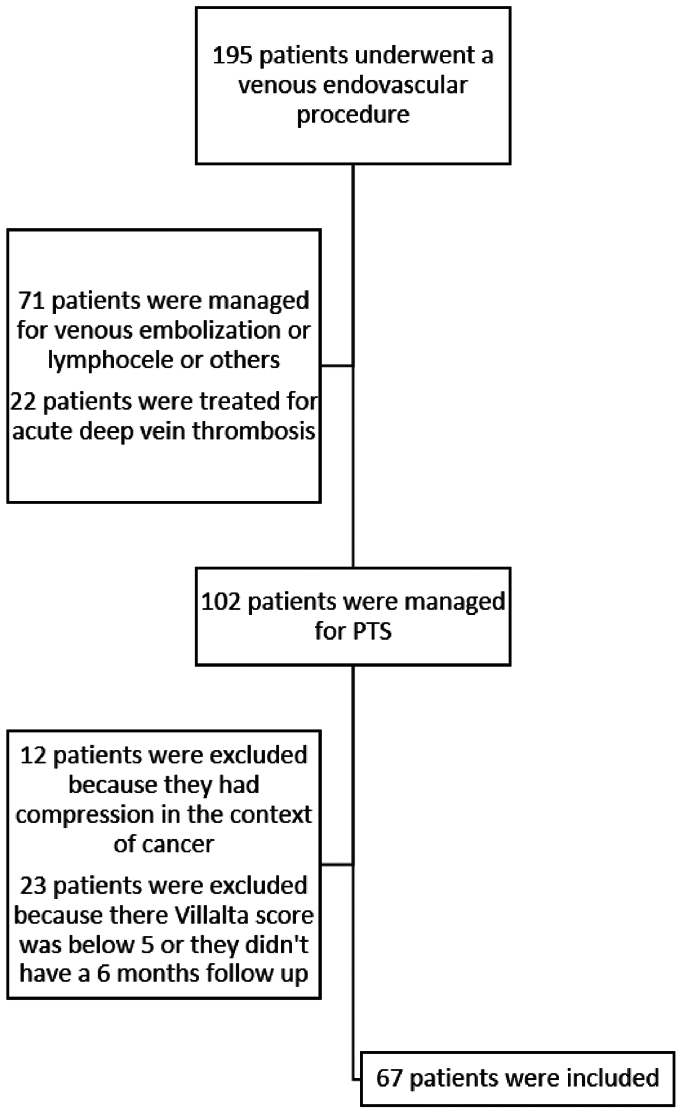
Flow chart. *PTS*, post-thrombotic syndrome.

Between January 1, 2015, and September 30, 2019, 67 consecutive patients were included (Fig 1) with median age of 40 years (IQR, 32-51 years), among whom were 52 women (78%) and 12 with obesity (18%) (Table I). The median follow-up was 16.9 months (IQR, 7.9-24.6 months). The median time from last DVT to venous recanalization was 24.8 months (IQR, 12.2-103.1 months), with 21 patients (31%) with 2 episodes of DVT, 5 patients (8%) with ≥3 episodes, and 10 patients (15%) with a known severe thrombophilia. The initial Villalta score was 10 (IQR, 7-14) and 36 patients (54%) had moderate or severe PTS (Supplementary Table I, online only). The extension of venous obstruction was limited to the common femoral and iliac veins in 41 patients (61%) and included the popliteal, femoral, common femoral and iliac veins in 22 patients (33%) (Supplementary Table II, online only). Six patients (9%) had superficial venous insufficiency. Among the 21 patients (31%) treated with long-term therapeutic anticoagulation at the time of the procedure, one-half had a history of at least two DVT episodes and their initial Villalta score was significantly higher (median, 8; IQR, 6-12) compared with those without long-term anticoagulation (median, 4; IQR, 9-16; *P* = .001).

**Table I:** Baseline characteristics of patients

Included patients (n = 67)	
Female sex	52 (77.6)
Age, years	40 [32-51]
BMI, kg/m^2^	24.2 [20.7-28.3]
Obesity (BMI ≥30 kg/m^2^)	12 (17.9)
History of chronic venous disease	6 (9.0)
Long-term therapeutic anticoagulation	21 (31.3)
Thrombophilia	
Antithrombin deficiency	0/45 (0)
Protein C deficiency	3/44 (6.8)
Protein S deficiency	2/44 (4.5)
Antiphospholipid syndrome	4/66 (6.1)
Heterozygous Factor II G20210 A mutation	5/46 (10.9)
Heterozygous Factor V Leiden mutation	8/46 (17.4)
Homozygous or double heterozygous FII G20210 A mutation and/or FV Leiden mutation	1/46 (2.2)
Venous obstruction localization	
Inferior vena cava	1 (1.5)
Common iliac vein	48 (71.6)
External iliac vein	58 (86.6)
Common femoral vein	40 (59.7)
Femoral vein	25 (37.3)
Deep femoral vein	3 (4.5)
Popliteal vein	9 (13.4)
PTS	
Villalta score	10 [7-14]
PTS severity according to Villalta score	
Mild (5-9)	31 (46.3)
Moderate (10-14)	24 (35.8)
Severe (≥15)	12 (17.9)
Ulcer	7 (10.4)

*BMI,* Body mass index; *DVT,* deep venous thrombosis; *PTS,* post-thrombotic syndrome.

Values are number (%) or median [interquartile range].

### Procedure characteristics and antithrombotic therapy

Immediate procedural success was achieved in all cases (100%). The median number of stents was 2 (IQR, 2-3), and the most frequently implanted sites were the external iliac vein, the common iliac vein, and the common femoral vein (Table II). The primary patency rate was 85% (57/67) at day 7 and 75% (50/67) at 6 months. A secondary procedure was successful in 6 of 10 patients with occlusion at day 7. The secondary patency rate was 94% (63/67) at day 7 and 81% (54/67) at 6 months. Stent thrombosis at 6 months was associated with a higher Villalta score at baseline despite a Villalta score decrease at 6 months (Supplementary Table III, online only).

**Table II:** Procedure characteristics

Included patients (n = 67)	
No. of stents	2 [2-3]
1	4/61 (6.6)
2	30/61 (49.2)
3	20/61 (32.8)
4	5/61 (8.2)
5	2/61 (3.3)
Stent localization	
Inferior vena cava	3 (4.5)
Common iliac vein	54 (80.6)
External iliac vein	57 (85.1)
Common femoral vein	48 (71.6)
Femoral vein	9 (13.4)
Deep femoral vein	1 (1.5)
Popliteal vein	1 (1.5)
Immediate postprocedure therapeutic anticoagulation	67 (100)
DOAC	52 (77.6)
Low molecular weight heparin	14 (20.9)
UFH	1 (1.5)
Immediate postprocedure antiplatelet therapy	61 (91.0)
Aspirin	46 (68.7)
Clopidogrel	15 (22.4)
Immediate postrecanalization complications	
None	64 (95.5)
Death	0 (0.0)
Major bleeding	0 (0.0)
Non-major but clinically relevant bleeding	1 (1.5)
Minor bleeding	1 (1.5)

*DOAC,* Direct oral anticoagulant; *UFH,* unfractionated heparin.

Values are number (%) or median [interquartile range].

Antithrombotic therapy included therapeutic anticoagulation in all patients with either a DOAC (77.6%), low-molecular-weight heparin (20.9%), or UFH (1.5%) and antiplatelet therapy in 61 patients (91%). No (0.0%) major bleeding but one clinically relevant nonmajor bleeding (arteriovenous fistula at the puncture site requiring reintervention) and one minor bleeding (hematoma at the puncture site) occurred just after the procedure.

At 6 months, 32 patients (48%) were receiving therapeutic anticoagulation alone, 6 (9%) antiplatelet therapy alone, 19 (28%) both therapies, and 10 (15%) no antithrombotic therapy. Antiplatelet therapy was stopped after a median of 174 days (IQR, 94-206 days), and therapeutic anticoagulation after a median of 198 days (IQR, 179-284 days). No (0.0%) major bleeding but one additional minor bleeding (abnormal uterine bleeding) occurred within 6 months. At the last visit, 47 patients (70%) were being treated with therapeutic anticoagulation.

### Improvement of Villalta score over time

**Fig 2:**
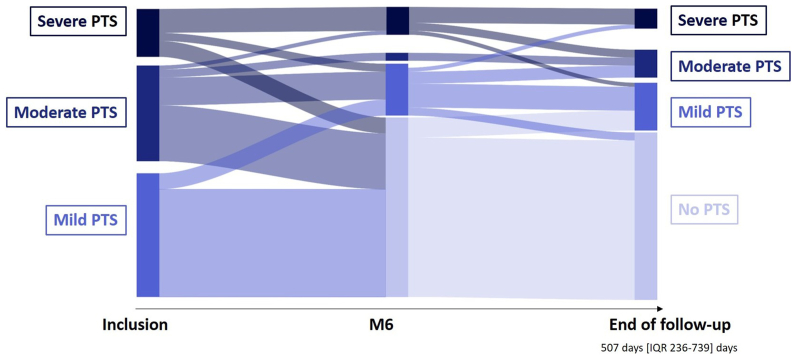
Sankey diagram of post-thrombotic syndrome (*PTS*) severity change during follow-up after endovascular procedures.

At 6 months, the median decrease in the Villalta score was 6 points (IQR, 3-8 points). Forty-five patients (67%) had no PTS with a median decrease in the Villalta score of 7 points (IQR, 5-10 points), leading to a median Villalta score of 2 points (IQR, 0-3 points). The 22 other patients (33%) had a less of a median decrease in the Villalta score: 2 points (IQR, 0-6 points; *P* < .001) leading to a Villalta score of 9 (IQR, 6-15; *P* < .001) (Table III and Fig 2). At the last visit follow-up, 40 patients (60%) had no PTS. Among the seven ulcers initially present, two healed within 6 months and two others during follow-up at 353 and 551 days. The three patients with nonhealed ulcers had an occluded stent early after the initial procedure.

**Table III:** Post-thrombotic syndrome (*PTS*) evaluation at 6 months

	No PTS (n = 45)	PTS (n = 22)	*P* value
Baseline clinic characteristics			
Female sex	8 (17.8)	7 (31.8)	.22
Age, years	39.0 [30.0-49.5]	42.5 [34.0-55.8]	.12
BMI, kg/m^2^	24.1 [20.5-28.0]	24.6 [20.9-30.1]	.52
Obesity (BMI ≥30 kg/m^2^)	6 (13.3)	6 (27.3)	.19
Baseline Villalta score	8.0 [6.0-12.0]	14.0 [11.0-15.0]	<.001
Mild PTS	27 (60.0)	4 (18.2)	
Moderate PTS	14 (31.1)	10 (45.5)	
Severe PTS	4 (8.9)	8 (36.4)	
Ulcer	2 (4.4)	5 (22.7)	.034
Long-term anticoagulation indication	10 (22.2)	11 (50.0)	.021
Baseline ultrasound characteristics			
Femoral-popliteal obstruction	4 (8.9)	7 (31.8)	.032
Femoral obstruction	13 (28.9)	12 (54.5)	.041
Femoral-popliteal reflux	0 (0.0)	2 (9.1)	.10
Baseline biological characteristics			
Severe thrombophilia	2/45 (4.4)	2/21 (9.5)	.59
Nonsevere thrombophilia	12 (26.7)	5/21 (23.8)	.81
Protein S deficiency	0/30	2/14 (14.3)	.10
Protein C deficiency	1/30 (3.3)	2/14 (14.3)	.23
C-reactive protein, mg/L	1.0 [1.0-1.8]	3.0 [1.0-6.8]	.027
Leucocytes, G/L	7.8 [6.7-9.0]	6.1 [4.9-8.7]	.025
Clinical evaluation at 1 month			
Early stent occlusion (within 7 days)	3 (6.7)	7 (31.8)	.011
Persistence of PTS	3/37 (8.1)	10/17 (58.8)	<.001
Early femoral vein occlusion	2 (4.4)	6 (27.3)	.013
Villalta Score	2 [1-3]	7 [3-10]	.001
Clinical evaluation at 6 months			
Secondary patency rate	42 (93.3)	12 (54.5)	<.001
Femoral vein occlusion	2 (4.4)	9 (40.9)	<.001
Ulcer	0 (0.0)	5 (22.7)	.003
Villalta score	2 [0-3]	9 [6-15]	<.001
Villata score reduction from baseline	7 [5-10]	2 [0-6]	<.001

*BMI,* Body mass index.

Values are number (%) or median [interquartile range].

### Predictive factors of PTS at 6 months

Patients with PTS 6 months after the procedure presented a higher initial Villalta score (*P* < .001) and more frequently a venous ulcer (*P* = .034) compared with patients without PTS (Table III). Femoral vein obstruction at baseline was also associated with PTS (*P* = .041), but not sex, age, or obesity. Patients with PTS presented a higher Villalta score at 1 month (median, 7 [IQR, 3-10] vs 2 [IQR 1-3]; *P* = .001), a lower secondary patency rate (55.0% vs 93.0%; *P* < .001), with more stent occlusion within 7 days (31.8% vs 6.7%; *P* = .011) compared with patients without PTS. However, neither the number of stents nor their localizations were associated with the persistence of PTS. Antithrombotic treatments at 6 months did not differ between groups.

Using multivariate analysis of initial characteristics, the initial Villalta score (OR, 1.36; 95% CI, 1.12-1.65; *P* = .002), femoral vein obstruction (OR, 3.79; 95% CI, 1.06-13.61; *P* = .041) but not long-term therapeutic anticoagulation (OR, 1.37; 95% CI, 0.36-5.22; *P* = .643) were predictive of persistent PTS. We then computed a risk score by adding 4 in case of femoral vein obstruction to the initial Villalta score. The weight of 4 points attributed to femoral vein sequelae in the score was derived from the OR observed in the multivariate analysis (OR, 3.79). This value was approximated for simplicity and proportionality, using the same rationale as the incremental risk per Villalta point (OR of 1.36 ≈ 1 point). The risk score was higher in patients with PTS (15; IQR, 12-19) compared with patients with no PTS (10; IQR, 7-13; *P* < .001). A risk score of <10 points had 96% sensitivity and 40% specificity to predict no PTS at 6 months, whereas a risk score of ≥15 points had 50% sensitivity and 88% specificity to predict persistent PTS at 6 months.

## Discussion

This study provides predictive factors of persistent PTS 6 months after endovascular venous recanalization. This technique can free two-thirds of patients from PTS, but those with a high Villalta score and sequelae of the femoral vein at baseline are at risk of persistent PTS at 6 months despite the procedure. There is controversial evidence regarding the use of GCS for the treatment of PTS.^34^, ^35^, ^36^ One randomized controlled trial reported beneficial hemodynamic effects, whereas another one found no benefit on PTS severity compared with placebo stockings.^37^^,^^38^ Although all patients had persistent symptoms despite initial compression therapy in our study, it remains conceivable that those with lower Villalta scores may have had a more favorable response to postprocedural GCS, contributing to better outcomes.

The magnitude of benefit likely varies with baseline severity.^14^ Physical exercise has a beneficial impact in muscle function and strength allowing to improvement of PTS symptoms and patient quality of life.^39^^,^^40^ Recently, ATLANTIS (Aquatic Therapy to Lower Adverse CoNsequences of Venous Thrombosis and InSufficiency) reported that supervised aquatic activity resulted in a 4-point decrease of the Villalta score at 2 years.^41^ In our cohort a median reduction of 6 points of the Villalta score was obtained 6 months after venous recanalization. More interestingly, the magnitude was greater in patients who recovered from their PTS with a 7-point decrease compared with the 2 points in those with persistent PTS. Therefore, the initial severity of PTS is not only predictive of the PTS recovery, but also of the magnitude of the benefit of the intervention. For comparison, a decrease in the Villalta score of 7.8 points at 6 months after proximal vein recanalization was reported in ACCESS PTS (Accelerated Thrombolysis for Post-Thrombotic Syndrome Using the Acoustic Pulse Thrombolysis Ekosonic Endovascular System), a 6-point decrease at 6 and 12 months by Black et al and an 8.4-point decrease at 12 months in TOPOS (Treatment of the Postthrombotic Syndrome With the Oblique Stent).^16^^,^^27^^,^^42^ Besides, 60% of patients were free from PTS at 12 months in TOPOS, whereas Black et al reported a 33% and 29% rate at 6 and 12 months, respectively.^27^^,^^42^ Among the predicting factors associated with persistent PTS 6 months after venous recanalization, obstruction of the femoral vein represented a risk for occlusion of the femoral vein after the procedure, even though neither the number nor the localization of stents were associated with persistent PTS. Persistent occlusion of the femoral vein is responsible of less inflow and create a persistent hemodynamic occlusion. In addition, the position under the inguinal ligament of the stents represents a risk factor for restenosis and stent fracture.^24^^,^^43^

We did not face stent fracture and the rate of stent crossing the inguinal ligament in our series (72%) was similar to the rate reported in TOPOS (70%).^27^ Moreover, 78% of our patients were on a DOAC. As shown in a study comparing the safety and efficacy of rivaroxaban vs warfarin for patients with PTS undergoing iliofemoral venous stenting, the primary patency rate was higher in the rivaroxaban group at 1 year.^44^ With a 75% primary patency and an 81% secondary patency rates at 6 months, our results are in line with previous studies.^21^^,^^45^^,^^46^ In addition, at the end of the follow-up of 16.9 months, the secondary patency rate was 83%, which is comparable with the 90% after 2 years of follow-up reported by Rosales et al.^47^ In contrast, early occlusions (within the first 7 days; 15%) in our series appeared to be slightly higher than the 10% usually reported.^48^^,^^49^ This finding could be explained by the absence of intravascular ultrasound examination at the beginning of our local experience, which could detect stent mispositioning at the end of the procedure, as described by Ginsberg et al.^37^ Our policy of systematic desobstruction by pharmacomechanical thrombectomy and repeat stenting allowed to recover many of these early occlusions. Similarly to Gwozdz et al,^50^ we did not find different outcomes in patients with thrombophilia compared with those without, suggesting that these patients should probably not be excluded from this type of procedure.

The combination of antiplatelet therapy with anticoagulant therapy was shown in a retrospective analysis to be predictive for stent patency.^51^ In our series, all patients received anticoagulant, 91% received antiplatelet therapy after the procedure, and 85% had at least one antithrombotic at 6 months, which is in line with other studies.^52^ The bleeding risk despite this dual antithrombotic strategy seemed to be low; no major hemorrhagic events perioperatively or at 6 months were observed. Qiu et al's meta-analysis^21^ showed similar result, with a 3.4% complication rate within the first 30 days but no major bleeding events. These results support the dual antithrombotic strategy when there is no increased risk of bleeding as recommended by the American Heart Association.^53^

Our study has several limitations, among which is the nonrandomized retrospective nature of this analysis. However, the homogeneity of the patients included is also one of its strengths: all had a confirmed PTS with obstruction on duplex ultrasound, all had a follow-up of ≥6 months, with Villalta score assessment, and none had nonthrombotic iliac vein lesions, as in some other studies.^54^ The lack of a control group is another limitation. A Dutch study showed that the incidence of PTS was quite stable 1 year after DVT, with 22% at 1 year and 29% at 8 years.^55^ The benefit on PTS that we report herein addressed patients with a delay from DVT of 2 years. Their PTS was consequently entrenched, and the natural improvement probably low, making a control group futile. That delay was longer than other series, which included patients with a mean time of 108 days at most from DVT, which strengthens the benefit of venous recanalization in entrenched PTS.^21^^,^^47^ Our decision to include patients with Villalta scores between 5 and 9 is consistent with French current expert consensus, which acknowledges that the Villalta score does not encompass all clinically relevant features of PTS, such as venous claudication. According to recent recommendations from the French Society of Vascular Medicine and the Cardiovascular French Society of Radiology, venous recanalization may be considered in patients with mild PTS if symptoms significantly impact quality of life.^30^ Our study did not include an assessment of quality of life like the CIVIQ-20 (a chronic lower limb venous insufficiency questionnaire), Clinical, Etiological, Anatomical, Pathophysiological classification, or the Venous Clinical Severity Scores, because these scores were not specifically developed to assess PTS. Yet, the Villalta score alone can also be questioned in the evaluation of PTS severity. International recommendations suggest treating patients with iliac vein flow obstruction with recalcitrant venous ulcers, severe PTS, or disabling venous claudication. Venous claudication, usually described as heaviness and pain on exertion that subsides at rest, frequently qualify patients with mild or moderate PTS. All our patients have been managed in multidisciplinary team as recommended and were discussed at a staff meeting to decide on the benefit of intervention. Intravascular ultrasound examination was not used systematically but liberally in all cases when the immediate venographic result was not optimal. Finally, we could construct a risk score by computing the initial Villalta score with addition of 4 points for femoral vein sequelae. We acknowledge the very preliminary approach of this score, which is more to provide a better understanding of the risk factors for persistent PTS but would require a validation on a replication cohort before its implementation in clinical practice.

## Conclusions

Our study has shown that venous recanalization with stenting can free two-thirds of patients from PTS 6 months after procedure, especially those with a low Villalta score and no sequelae of the femoral vein at baseline. A risk score based on these parameters could be an additional aid for the clinician in providing information to the patient on the expected benefits of venous recanalization.

## Author contributions

Conception and design: LK, GG, EM, MS, TM

Analysis and interpretation: LK, NG, CG, MA, TM

Data collection: LK, NG, MA, MS, TM

Writing the article: LK, NG, TM

Critical revision of the article: LK, CDG, NG, CG, MA, GG, EM, MS, TM

Final approval of the article: LK, CDG, NG, CG, MA, GG, EM, MS, TM

Statistical analysis: TM

Obtained funding: Not applicable

Overall responsibility: LK

MS and TM equally contributed to this work and share senior authorship.

## Funding

None.

## Disclosures

L.K., N.G., and T.M. acknowledge the following without any relation with the current manuscript. L.K. reports receiving honoraria from Bristol-Myers Squibb, Viatris, and Pfizer SAS and receiving nonfinancial support from Bristol-Myers Squibb, Viatris, Pfizer SAS, Leo Pharma, and Sanofi. N.G. discloses consulting fees or travel awards by Bayer, Bristol-Myers Squibb/Pfizer, and LEO-Pharma. T.M. reports receiving honoraria from Bayer Healthcare SAS and Incyte Biosciences France and receiving nonfinancial support from Abbott France, Alexion Pharma France, Amgen SAS, Bayer Healthcare SAS, Boehringer Ingelheim France, Bristol-Myers Squibb, ICOMED, Incyte Biosciences France, MSD France, and Pfizer SAS.
