# Supplementary material for: Predictive factors for complete recovery of post-thrombotic syndrome 6 months after venous recanalization
Source: J Vasc Surg Venous Lymphat Disord. 2025 Jul 11;13(6):102287. doi: 10.1016/j.jvsv.2025.102287 (PMC12358624; doi:10.1016/j.jvsv.2025.102287)
Supplement: Supplementary Materials [file mmc1.docx]

**Supplementary material for “Predictive Factors for Complete Recovery of Post-thrombotic Syndrome 6 Months after Venous Recanalization”**

Lina Khider, Costantino Del Giudice, Nicolas Gendron, Chloé Gobert, Benjamin Planquette, Marc Al Ahmar, Guillaume Goudot, Emmanuel Messas, Marc Sapoval, Tristan Mirault

**Supplementary Table 1. Characteristics of patients with mild versus moderate or severe PTS at baseline.**

IQR: interquartile range. PTS: post-thrombotic syndrome, DVT: deep venous thrombosis, BMI: body mass index; Het: heterozygous.

| **Patients** | **Mild PTS**  **(n=31)** | **Moderate or Severe PTS (n=36)** | **p-value** |
| --- | --- | --- | --- |
| Female sex | 26 (83.9) | 26 (72.2) | .38 |
| Age – years, median [IQR] | 38.5 [30.0–49.5] | 44.0 [37.5–54.5] | .027 |
| BMI – kg/m², median [IQR] | 23.0 [20.3–27.1] | 26.4 [21.8–29.4] | .11 |
| Obesity (BMI ≥ 30kg/m²) | 3 (9.7) | 9 (25.0) | .12 |
| History of chronic venous disease | 3 (9.7) | 3 (8.3) | 1.00 |
| Long-term therapeutic anticoagulation | 6 (19.4) | 15 (41.7) | .05 |
| **Thrombophilia** | | | |
| Antithrombin deficiency | 0 (0.0) | 0 (0.0) | 1.00 |
| Protein C deficiency | 1 (4.3) | 2 (9.5) | .60 |
| Protein S deficiency | 0 (0.0) | 2 (9.5) | .22 |
| Het. Factor II G20210A mutation | 3 (13.0) | 3 (13.0) | 1.00 |
| Het. Factor V Leiden mutation | 6 (26.1) | 3 (13.0) | .46 |
| Antiphospholipid syndrome | 2 (9.7) | 2 (5.6) | .33 |
| **Venous obstruction localization** | | | |
| Inferior vena cava | 0 (0.0) | 1 (2.8) | 1.00 |
| Common iliac vein | 21 (67.7) | 27 (75.0) | .59 |
| External iliac vein | 27 (87.1) | 31 (86.1) | 1.00 |
| Common femoral vein | 19 (61.3) | 21 (58.3) | 1.00 |
| Femoral vein | 13 (41.9) | 12 (33.3) | .61 |
| Deep femoral vein | 1 (3.2) | 2 (5.6) | 1.00 |
| Popliteal vein | 4 (12.9) | 5 (13.9) | 1.00 |
| Initial Villalta | 7 [6–8] | 12 [11–15] | < .001 |
| **Clinical evaluation at 1 month** | | | |
| Early stent occlusion (within 7 days) | 4 (12.9) | 6 (16.7) | .74 |
| Persistance of PTS | 1 (3.8) | 12 (42.9) | .001 |
| Villalta Score, median [IQR] | 2 [1–3] | 4 [1–8] | .08 |
| **Clinical evaluation at 6 months** | | | |
| Persistance of PTS | 4 (12.9) | 18 (50.0) | .002 |
| Villalta Score, median [IQR] | 2 [0–4] | 3 [2–9] | .001 |

**Supplementary Table 2. Extension of venous obstruction at baseline and outcome at 6 months after revascularization.**

We used the LET classification which was designed to identify patients at high risk for developing post-thrombotic syndrome (PTS) in the acute phase using thrombus location and extent with a prospective validation.[1,2] Although not developed in chronic venous obstruction, the distribution according to the LET classification of the venous obstruction at baseline is presented in the **supplementary table 2 and 3.** Villalta score at baseline did not differ according to LET class: LET II Villalta 11 [7–14]; LET II+III Villalta 9 [7–14]; LET III Villalta 10 [7–13] (p = 1.00). Finally, there was no significant difference (p = .09) in the distribution of the LET classes according to the absence or presence of PTS at 6 months.

Results are number (percentage). PTS: post-thrombotic syndrome, LET: lower extremity thrombosis, LET Class I: calf veins; Class II: popliteal and femoral veins; Class III common femoral and iliac veins; Class IV: inferior vena cava.

|  | **All patients** | **No PTS** | **PTS** | **p-value** |
| --- | --- | --- | --- | --- |
| **Patients** | **n = 67** | **n = 45** | **n = 22** |  |
| **LET class** |  |  |  | .09 |
| I+II | 1 (1.5%) | 0 (0.0%) | 1 (4.5%) | - |
| II | 2 (3.5%) | 1 (2.2%) | 1 (4.5%) | - |
| II+III | 22 (32.9%) | 12 (26.7%) | 10 (45.5%) | - |
| III | 41 (61.1%) | 32 (71.1%) | 9 (40.9%) | - |
| III+IV | 1 (1.5%) | 0 (0.0%) | 1 (4.5%) | - |

**Supplementary Table 3. Characteristics of patients with stent thrombosis versus patent stent at 6 months after revascularization.**

Results are median [interquartile range (IQR)] or number (percentage). LET: lower extremity thrombosis, LET Class I: calf veins; Class II: popliteal and femoral veins; Class III common femoral and iliac veins; Class IV: inferior vena cava.

| **Patients** | **Patent stent at 6 months, n=54** | **Stent thrombosis at 6 months, n=13** | **p-value** |
| --- | --- | --- | --- |
| Villalta score at baseline | 9.5 [6.8–12.3] | 14.0 [7.5–16.0] | .029 |
| Villalta score at 6 months | 2.0 [0.8–4.0] | 8.0 [4.5–15.0] | .001 |
| LET Class |  | | .22 |
| I+II & II | 1 (1.9%) | 2 (15.4%) |  |
| II+III & III | 52 (96.3%) | 11 (84.6%) |  |
| III+IV | 1 (1.9%) | 0 (0.0%) |  |

**References**

[1] C.W.K.P. Arnoldussen, C.H.A. Wittens, An imaging approach to deep vein thrombosis and the lower extremity thrombosis classification, Phlebology 27 Suppl 1 (2012) 143–148. https://doi.org/10.1258/phleb.2012.012s25.

[2] R.H.W. Strijkers, C.W.K.P. Arnoldussen, C.H.A. Wittens, Validation of the LET classification, Phlebology 30 (2015) 14–19. https://doi.org/10.1177/0268355515569133.
